# Legume-maize rotation effect on maize productivity and soil fertility parameters under selected agronomic practices in a sandy loam soil

**DOI:** 10.1038/s41598-019-43679-5

**Published:** 2019-06-12

**Authors:** Ifeyinwa Monica Uzoh, Charles Arizechukwu Igwe, Chinyere Blessing Okebalama, Olubukola Olularanti Babalola

**Affiliations:** 1 Food Security and Safety Niche Area, Faculty of Natural and Agricultural Science, North-West University, Private Bag X2046, Mmabatho, 2745 South Africa; 2 Department of Soil Science, Faculty of Agriculture, University of Nigeria, Nsukka Road, Nsukka, 410001 Nigeria

**Keywords:** Plant ecology, Environmental impact

## Abstract

Inclusion of legumes in cropping systems is essential for sustainable management of farming systems and reducing the nitrogen (N) fertilizer requirement for maize production. The study evaluated the effect of growing legumes (soybean, cowpea and velvet bean) and maize the same year in rotation, on maize yield and soil fertility indices. The agronomic practices implemented were residue management (residue added and residue removed) and fertilizer N application (0 kg N ha^−1^ and 60 kg N ha^−1^) under four rotation systems. The result showed that growing velvet bean the same year in rotation with maize was effective in increasing maize yield and improving some soil fertility indices over growing maize after maize the same year in the same location. Compared to maize monocropping, over 100% increase in maize yield was obtained with velvet bean-maize rotation even in absence of residue incorporation. In addition, velvet bean-maize rotation increased maize yield over cowpea- and soybean- maize rotations. The rotation effect occurred as a result of improvement in soil nitrogen, avail phosphorus (P), exchangeable magnesium (exch Mg) and effective cation exchange capacity (ECEC). Grain legumes-maize rotations equally increased maize yield over sole maize. Generally legume-maize rotations increased total N, avail P, exch K, Mg and effective cation exchange capacity over sole maize. Crop residue incorporation and N fertilizer application significantly improved soil N and maize grain yield (0.18%, 2.74 tha^−1^ in 2008; 0.22%, 1.16 tha^−1^ in 2009 and 0.19%, 2.72 tha^−1^ in 2008; 1.35 tha^−1^ in 2009 respectively) over non-residue incorporation (0.16% and 1.84 tha^−1^ in 2008, 0.66 tha^−1^ in 2009) and zero N application (0.16% and 1.83 tha^−1^ in 2008 and 0.17% and 0.85 tha^−1^ in 2009). Therefore, velvet bean could be planted the same season with subsequent maize in rotation cropping for intensive sustainable maize production in sandy-loam soils without fertilizer N. For grain legumes such as soybean and cowpea to be effective in rotation cropping with maize, the grain legumes have to be planted early before the full set of rain because excess rain would affect their growth and development.

## Introduction

Intensive maize production requires a lot of fertilizer N, which is expensive and inaccessible to resource poor farmers. More so, production of N fertilizers is high energy consuming^1^ and its use in farming is regarded as unsustainable. Therefore, sustainable management systems for intensive maize production are sought. Crop rotation affects the sustainability of agricultural production systems^2–4^. Often, crop rotations involve legumes since they are capable of fixing nitrogen. It is noted that legumes play a vital role in having beneficial food secure systems^5^, and rotations involving legumes diversify crop production systems and improve fertility of soils^6^. In their study of the effect of herbaceous legumes on subsequent crop, Entz, *et al*.^7^ stated that the accumulated nutrient in the residue green manure biomass increased grain yields of subsequent maize grown on legume plots. Their study also reported that the removal of legume residue during the dry season didn’t rule out the yield response of maize, but the magnitude of response depended on the fertility level of the location.

Another important aspect of residue management is the quality. Residue quality determines the effect of legumes on productivity of soils either by affecting availability of nutrients or soil organic matter. The effect on nutrient availability occurs through release of nutrients or indirectly through decomposition products (organic ions). Legume crop residues after mineralization by soil microbes, release available N for the subsequent crop^8–10^ and increase the nutrient status of the soil. It is well known that legumes do add organic N and their biomass improve soil properties and activities of microorganisms. However, agriculturists believe that the resultant subsequent crop improvement will differ depending on prevalent legume system^4,11^. Green manure crops when incorporated into the soil are known for their improvement of soil properties^12^ and yield of crop grown after them^13^ but the question is can maize be sustainably grown after legumes the same year and on the same piece of land for sustainable intensification of maize production? And is it possible to sustainably grow maize after grain legumes such as soybean and cowpea? This could enable faster adoption of legume-maize rotations on farmers’ fields. Since annual grain legumes are not only used as green manure crops but produce grains that add economic return to farmers.

Seymour, *et al*.^13^ observed that cereal yield increase associated with legume was consistent over the years depending on location and legume crop used. The magnitude of rotation benefit provided by any legume depends on several factors such as the type of legume, its residue production and growing condition of the soil. It is important to evaluate this new crop management system of growing in the same year, two different crops in rotation. The hypothesis is that legume-cereal rotation with or without residue and/or N addition gives higher yield and improves soil fertility than maize monocrop. Alternative hypothesis is that legume-cereal rotation with or without residue and/or urea addition gives the same or lower yield and does not improves soil fertility than maize monocrop. So the study evaluated maize yield and soil fertility improvement of grain legumes-maize and velvet bean-maize rotations, with both crops grown the same year on the same piece of land under selected agronomic practices. The objectives were to; (1) determine the site specific effect of rotation benefit of soybean, cowpea and velvet bean cultivation on maize yield and soil fertility parameters and (2) determine the effect of residue management and N fertilizer as selected agronomic practices on moderating the rotation benefit to maize grain yield and soil chemical properties.

## Materials and Methods

### Description of site for the field experiment

The field experiment was established at Dominican Centre for Human Resources Development farm in Moniya, Oyo state, Nigeria. International Institute of Tropical Agriculture (IITA), Ibadan uses the centre for field experiments. Moniya is located between latitude 7°20′N and 7°28′N and longitude 30°50′E and 30°57′E. The average annual rainfall of 1119.55 mm with maximum and minimum average relative humidity of 90.81% and 48.88% in 2008 and 89.91% and 50.62% in 2009, respectively prevailed in the study area. The mean annual minimum and maximum temperature were 23 °C and 31 °C, respectively. The soil of Moniya was classified as a Plinthic Paleustalfs^14^, full of stone and dark brown to grey in colour.

### Characterization of soils of the study area

The soil’s physical and chemical characteristics of the study area shows (Table 1) that with the dominance of about 75% sand, and low silts (10%) contents, the soil was sandy loam. The pH was slightly acid. Cation exchange capacity, Ca, Mg, and K were moderate and BS was high. Total N was low (0.16%)^15^ but available P was moderate (4 mg kg^−1^). The area has been left fallow years before the conduct of the research so the area had relatively high nutrients especially the exchangeable cations.

**Table 1:** Soil properties at the beginning of the experiment.

Soil properties	Value
Clay (%)	15.9
Silt (%)	9.6
Sand (%)	74.6
Textural class	Sandy loam
pH	6.4
Total N (mg kg^−1^)	0.16
OC (mg kg^−1^)	1.39
C/N ratio	3.25
Avail. P (mg kg^−1^)	4.0
Exch. Ca (cmol kg^−1^)	4.04
Exch. Mg (cmol kg^−1^)	1.37
Exch. K (cmol kg^−1^)	0.4
Exch. Na (cmol kg^−1^)	0.16
Exch. Acid (cmol kg^−1^)	0.08
Effective CEC (cmol kg^−1^)	6.05
Apparent CEC (cmol kg^−1^)	12.5
Base Saturation (%)	98.7

### Experimental layout

This research was a rotational field trial that involved four cropping periods in two years. The experiment was conducted during the rainy season. In each year, maize, soybean, cowpea and velvet bean were planted on separate plots and allowed till maturity before they were harvested. Then after their harvest, maize was planted on all the plots to evaluate rotation benefit of previous crops on soil properties and yield of maize under two residue management (residue added and residue removed) and two N fertilizer rates (0 kg N ha^−1^ and 60 kg N ha^−1^). The design of the experiment design was 4*2*2 factorial in RCBD. This was replicated thrice to give a total of 48 plots.

### Field establishment and treatment application

Total area of 0.08 hectares was ploughed, harrowed and ridged and then demarcated into 3 blocks (replications), which were further divided each into 16 plots, totalling 48 plots. Each plot measured 3 m × 4.5 m. Seeds used for the experiment were obtained from gene bank of International Institute of Tropical Agriculture (IITA), Ibadan. Treatment combinations were assigned to each plot using randomization. Maize hybrid (Oba Super II), dual-purpose cowpea (03k-374-4), fast maturing soybean (TGX 1448) and velvet bean (*Mucuna pruriens*) at a spacing of 0.75 m × 0.25 m were planted, three seeds in each hole. These were thinned down to one seed per hole after two weeks of emergence. The same quantity of NPK fertilizer (15: 15: 15) was added 3 weeks after emergence in all the plots and the farm was weeded as appropriate. At maturity, the crops were harvested. The plots for residue application received all residue of the previous crop harvested from the plot and those for residue not applied, the entire residue was removed. Thereafter, for the second planting, maize seeds were planted three per hole in all the plots and thinned to one after two weeks of emergence. Plots for nitrogen application received 81 g of urea each, equivalent to 60 kg ha^−1^. The choice of 60 kg ha^−1^ was informed by the result of a previous green house study which showed that N at 60 kg ha^−1^ and 120 kg ha^−1^ were significantly (*P* ≤ 0.05) the same. The first planting in 2008 took place on 27–28 May while the second planting of maize to check rotation effect (after harvesting the first crops) on each of the plots was on 27–28 August. They were harvested on 18th November while the first and second planting in 2009 were undertaken on 12–14 June and 21 September. When the plants reached maturity in late November, they were harvested.

### Soil sampling and yield measurement

Topsoil samples were gotten from 0–15 cm over the entire plot using grid method and put together to obtain a composite sample before establishing the experiment. Samples were also collected after each harvest from individual treatment plots. The soil sample was dried under shade, passed through 2 mm sieve for subsequent chemical analyses [pH, organic carbon (OC), total N, exchangeable Ca, Mg, K, available P, ECEC and EA] and particle size distribution for the first soil sample collected at onset of the experiment. During the study, rainfall data for the study area were obtained from the GIS unit of IITA, Ibadan. Maize stubbles were harvested at maturity, oven-dried and weighed to obtain dry matter yield. The grains were removed from the cobs and weighed to obtain yield of the grain, which was converted to hectare with the following calculation;

1 $${Yield}({}^{\underline{t}}{h}{a})=\frac{{\rm{plot}}\,{\rm{yield}}\,\ast \,10000}{{\rm{plot}}\,{\rm{area}}}$$

### Analysis of samples in the laboratory

Samples were analyzed in analytical laboratory of IITA, Ibadan, Nigeria. Particle size distribution was determined by Bouyoucous hydrometer method^16^. Soil pH was determined using pH electrode meter by using 1: 2.5 soils to water mixture^17^; organic carbon (OC) by dichromate oxidation method^18^, while organic matter (OM) was gotten from the product of the value for organic carbon and “Van Berminelen factor” (1.724). Bases were extracted with 1 N ammonium acetate (NH_4_OAc) at pH 7 and K determined with flame photometer^19^, whereas atomic absorption spectrophotometer was used for reading exchangeable Ca and Mg in the extract. Using potassium chloride displacement method described by^20^ exchangeable acidity was determined. Summation of the exchangeable bases and exchangeable acidity gave ECEC. Using Bray I method, available P was extracted and the concentration of P in the extract was determined using spectrophotometer^20^. Using Micro-Kjeldahl digestion method^21^, soil total N was determined.

### Statistical analysis

The design of the experiment was 4 × 2 × 2 factorial in RCBD. Data gotten from this research were analysed for differences in variance (ANOVA) using software of statistical analysis system (SAS)^22^ to determine the main plot and interactions treatment effects. Mean separation was done by Fischer’s least significant difference (F-LSD) at probability level of 5%.

### Ethical approval

This article does not contain any studies with human participants or animals.

## Results and Discussion

### Effect of crop rotations, residue management and application of nitrogen on chemical properties of the soil

General over view of the ANOVA presented in Table 2 indicated that the crop rotation systems apart from Ca and K, influenced all the chemical soil properties significantly (*P* ≤ 0.05) after the first year planting period. Residue management significantly affected soil N, P and K while fertilizer N treatments affected soil N and P. The various interaction effects of the factors showed strong influence on soil P content than the other soil fertility parameters measured. The interaction between crop rotation systems and residue addition significantly affected soil pH, available P and EA in 2008 but influenced the EA and ECEC in 2009. Also in 2009, crop rotation systems and fertilizer N treatment affected only the soil N content. Specifically in Table 3, pH was significantly (*P* ≤ 0.05) decreased in velvet bean-maize rotation than the others. Remarkably, velvet bean-maize and soybean-maize rotations significantly increased mostly all the soil fertility parameters over cowpea-maize and continuous maize cropping, except in few cases, particularly the EA of which the highest value was obtained in maize monocropping. In the second year, however, velvet bean-maize rotation increased significantly the soil N. More so, significant increase in soil N was also recorded with fertilizer N at 60 kg ha^−1^ both in 2008 and 2009. In addition, the residue incorporation treatment increased significantly the soil N content of the soil in 2008 but in 2009, the soil pH was increased by the residue incorporation (6.83) over residues not incorporated (6.69). The positive impact of legume-cereal rotation on soil properties especially on soil total N was expected because research has shown that legumes increase soil N either because of fixed N or sparing effect since their demand for N is less than cereals^23^. Also, higher soil N and soil fertility parameters in velvet bean compared to cowpea and soybean could be associated with the quantity of biomass produced by each^24^. Production of dry matter depends on seasonal variations in weather conditions^25^. Soils grown previously with legumes had lower pH and this aligns with research results of Yan, *et al*.^26^, which states that cultivation of legumes increases acidity by proton release from their roots. This is further explained by^27^ that legumes release hydrogen ion from their roots by taking up cations. And so acidification is increased. Furthermore, Tang, *et al*.^28^ found positive correlation between released acid from roots of some legume pastures and excess cations contained in the shoot. And this was regressed to decline in soil exchangeable bases. Another explanation for lower pH during legume growth is because the consequence of nitrogen fixation is resultant acidification of the soil.

**Table 2:** Significant levels of effect of rotation cropping, residue incorporation and N application and their interaction on determined soil fertility parameters.

Treatment	df	pH	OC	N	P	Ca	Mg	K	EA	ECEC
**First year planting**										
Crop rotation (C)	3	*	*	**	***	ns	*	ns	***	***
Residue Management (R)	1	ns	ns	***	***	ns	ns	***	ns	ns
Fertilizer N application (F)	1	ns	ns	**	***	ns	ns	ns	ns	ns
C × R	3	***	ns	ns	***	ns	ns	ns	**	ns
R × F	1	ns	ns	ns	***	ns	ns	ns	ns	ns
C × F	3	ns	ns	*	***	ns	ns	ns	ns	ns
C × R × F	3	ns	ns	ns	***	ns	ns	ns	***	ns
**Second year planting**										
Crop rotation (C)	3	ns	ns	**	ns	ns	ns	ns	ns	ns
Residue management (R)	1	*	ns	ns	ns	ns	ns	ns	ns	ns
Fertilizer N application (F)	1	*	ns	*	ns	ns	ns	ns	ns	ns
C × R	3	ns	ns	ns	ns	ns	ns	ns	ns	ns
R × F	1	ns	ns	ns	ns	ns	ns	ns	ns	ns
C × F	3	ns	ns	ns	ns	ns	ns	ns	ns	ns
C × R × F	3	ns	ns	ns	ns	ns	ns	ns	ns	ns

Note *means significant at 5%, **highly significant at 1%, ***highly highly significant at 0% and ns means non-significant.

**Table 3:** Effect of legume-cereal rotation, residue incorporation and N rates on chemical properties of the soil.

Treatments	pH	OC %	N %	P mg kg^−1^	Ca	Mg	K	EA	ECEC
					cmol kg^−1^				
**2008**									
Velvet bean-Maize	6.17	1.49	0.19	7.70	1.75	1.0	0.24	0.08	3.18
Cowpea-maize	6.55	1.26	0.17	4.2	1.95	0.84	0.19	0.11	3.22
Soybean-Maize	6.53	1.50	0.19	6.9	1.93	0.84	0.21	0.12	3.19
Maize-maize	6.74	1.29	0.16	6.07	1.91	0.83	0.18	0.16	2.31
LSD (0.05)	0.28**	0.19*	0.02**	1.65**	ns	0.16*	ns	0.03**	0.59**
**N application**									
0 kg N ha^−1^	6.48	1.37	0.16	6.07	1.89	0.83	0.22	0.11	3.27
60 kg N ha^−1^	6.51	1.39	0.18	6.38	1.88	0.92	0.19	0.11	3.12
LSD (0.05)	ns	ns	0.01^**^	ns	ns	ns	ns	ns	ns
**Residue management**									
Residue incorporated	6.57	1.41	0.19	5.89	1.93	0.86	0.19	0.12	3.20
Residue not incorporated	6.43	1.37	0.16	6.56	1.85	0.9	0.22	0.11	3.19
LSD (0.05)	ns	ns	0.01^**^	ns	ns	ns	ns	ns	ns
Velvet bean-Maize	6.78	1.48	0.27	2009 12.38	3.14	1.19	0.31	0.03	4.83
Cowpea-maize	6.68	1.43	0.18	14.10	2.77	1.03	0.32	0.07	4.30
Soybean-Maize	6.78	1.51	0.19	9.31	2.92	1.12	0.31	0.10	4.52
Maize-maize	6.80	1.51	0.13	8.91	2.84	1.14	0.30	0.06	4.46
LSD (0.05)	ns	ns	0.06^**^	ns	ns	ns	ns	ns	ns
**Nitrogen application**									
0 kg N ha^−1^	6.82	1.41	0.17	12.57	2.91	1.13	0.32	0.05	4.54
60 kg N ha^−1^	6.69	1.40	0.22	9.79	2.92	1.11	0.30	0.08	4.51
LSD (0.05)	0.1*	ns	0.04*	ns	ns	ns	ns	ns	s
**Residue management**									
Residue incorporated	6.83	1.47	0.21	12.83	2.90	1.08	0.31	0.18	4.47
Residue not incorporated	6.69	1.49	0.18	9.52	2.94	1.16	0.31	0.17	4.58
LSD (0.05)	0.10^**^	ns	ns	ns	ns	ns	ns	ns	ns

*significant at 5%, **highly significant at 1%, ns- not significant.

Comparing velvet bean-maize rotations with the cowpea- and soybean-rotations, the difference on their effect on soil properties could possibly be as a result of their growth performance and residue production. Velvet bean did very well in the field, producing a lot of biomass whereas cowpea and soybean didn’t grow very well and produced scanty biomass and consequential poor effect on soil properties. The reason for their poor growth was possibly because they were planted when rains were high.

### Rainfall received during the study

**Figure 1:**
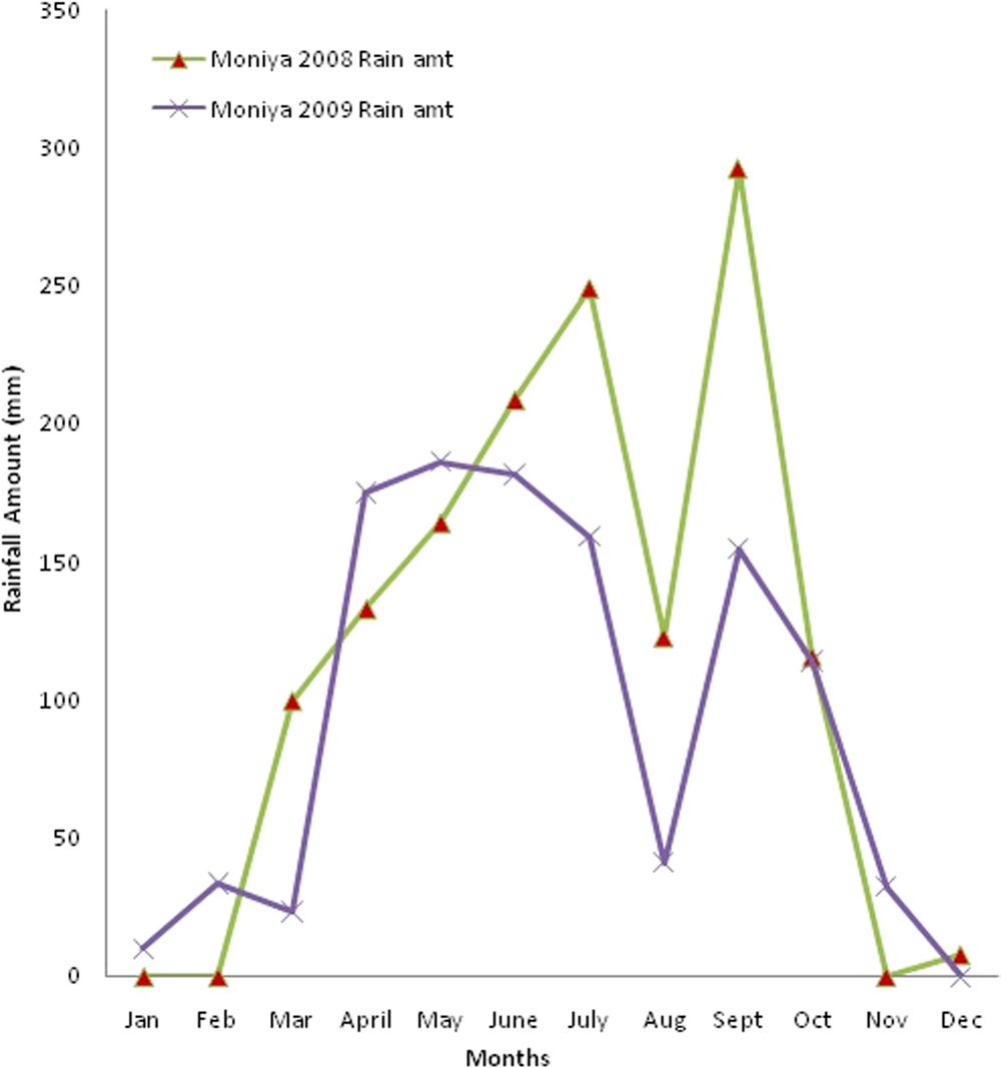
Rainfall amount for 2008 and 2009 at Moniya, Ibadan.

The monthly rainfall received at Moniya, Ibadan during the study period is shown in Fig. 1. In 2008, a total of 621.6 mm of rainfall was received during the first planting (May–July), which was excessive to the grain legumes so they performed badly but velvet bean performed well, producing plenty residue. The second maize received 531.1 mm of rain (August–October). In 2009, the second maize was planted in September and received a total of 301.75 mm of rain. By November, it rained only 5 days leading to reduction in growth period. Due to the high rainfall intensity especially in the first year, cowpea and soybean performance was poor when compared to velvet bean which had very luxurious biomass.

### Effect of legume-rotation systems and maize monocropping on the subsequent maize yield (rotation benefit)

**Figure 2:**
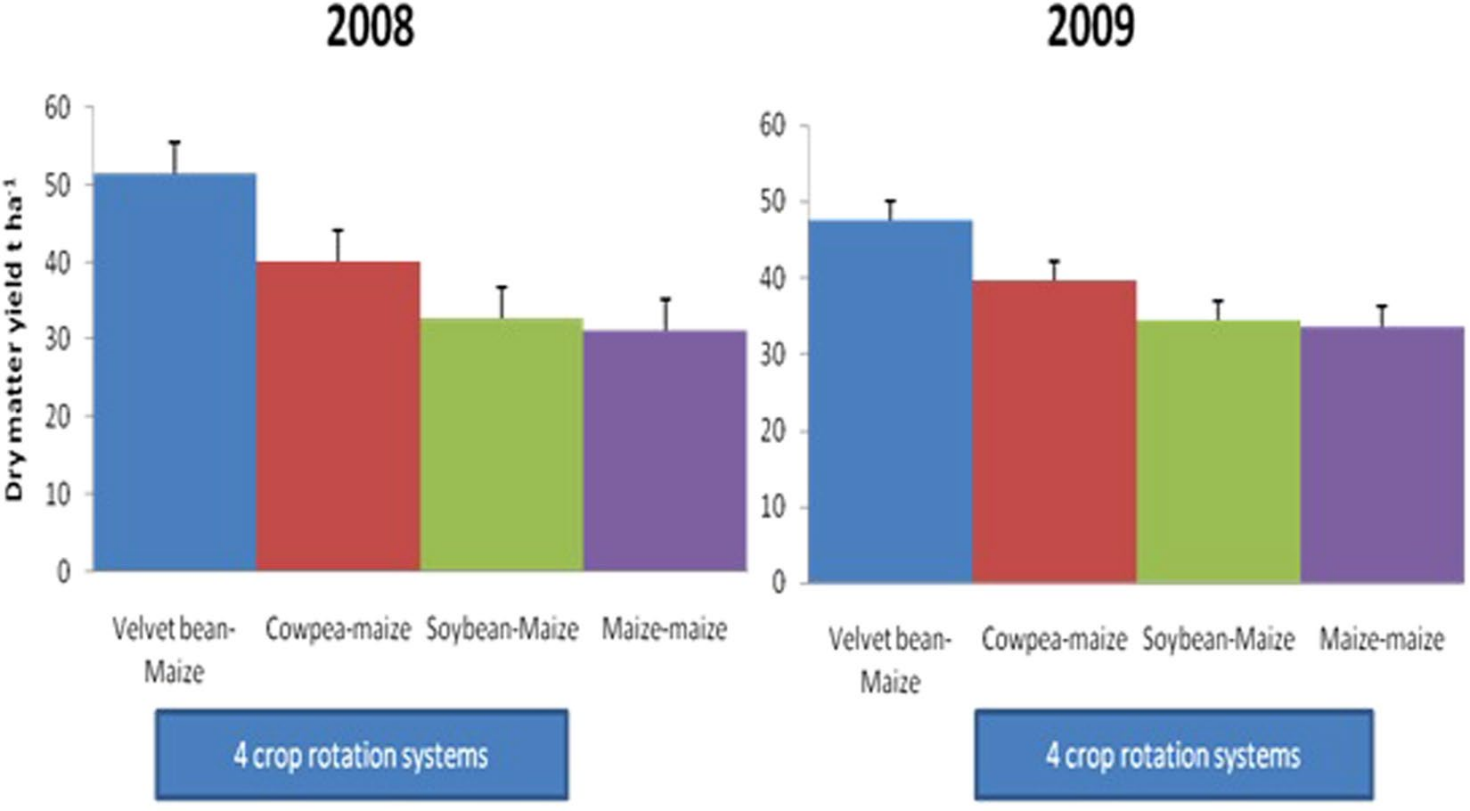
Effect of 4 crop rotation systems on maize dry matter yield in 2008 and 2009.

**Figure 3:**
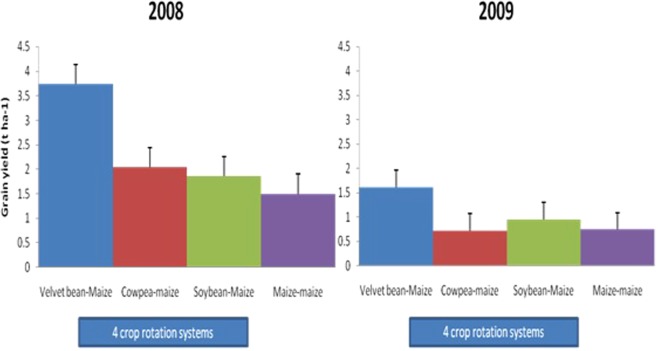
Effect of 4 crop rotation systems on maize grain yield in 2008 and 2009.

Maize yields were statistically higher in legume-cereal rotation plots (Table 4 and Figs 2 and 3). Among all legume rotation systems, plots previously grown with velvet bean gave significantly highest yields of maize sown in rotation in all the years; 3.74 tha^−1^ in 2008 and 1.61 tha^−1^ in 2009).

**Table 4:** Effect of crop rotation systems, residue application and N application rates on dry matter (t ha^−1^) and grain yield (t ha^−1^) of maize.

	Dry matter (t ha^−1^)		Grain yield (tha^−1^)	
	2008 Drymatter (t ha^−1^)	2009 Drymatter (t ha^−1^)	2008 Grain yield (tha^−1^)	2009 Grain yield (tha^−1^)
Velvet bean-Maize	51.47	47.59	3.74	1.61
Cowpea-maize	40.01	39.63	2.04	0.72
Soybean-Maize	32.75	34.44	1.86	0.95
Maize-maize	31.18	33.63	1.50	0.74
LSD (0.05)	4.01^**^	2.57^**^	0.40^**^	0.35^**^
**Nitrogen application**				
0 kg N ha^−1^	33.62	34.76	1.84	0.66
60 kg N ha^−1^	43.42	42.88	2.72	1.35
LSD (0.05)	1.05**	1.81**	0.28**	0.25**
**Residue management**				
No residue incorporated	35.02	35.68	1.83	0.85
Residue incorporated	42.02	41.96	2.74	1.16
LSD (0.05)	1.05**	1.82*	0.28**	0.25**

*Significant at 5% **highly significant at 1%, ns- not significant.

In 2009, the decline in the yield of maize could be attributed to less rain during the cultivation of the second maize (Fig. 1) and its growing period was shortened. The increase in the yield of maize rotated with velvet bean agrees with other findings. Maize grown after velvet bean is notable for increased yield^29^. In 2008, cowpea-maize rotation also increased the yield of maize significantly unlike soybean but in 2009 none of the grain legume-cereal rotations significantly increased yield of maize. Residue production by the grain legumes were poor due to excessive rainfall when they were planted, as a result their rotation benefit was non-significant. So effect of growing the grain legumes had little effect on the soil. That notwithstanding, the grain legumes-maize rotations still had higher yields compared to continuous maize. Velvet bean had luxurious growth with high biomass, thus gave significantly highest subsequent yield of maize.

### Effect of residue incorporation and N application in moderating the rotation benefit

**Figure 4:**
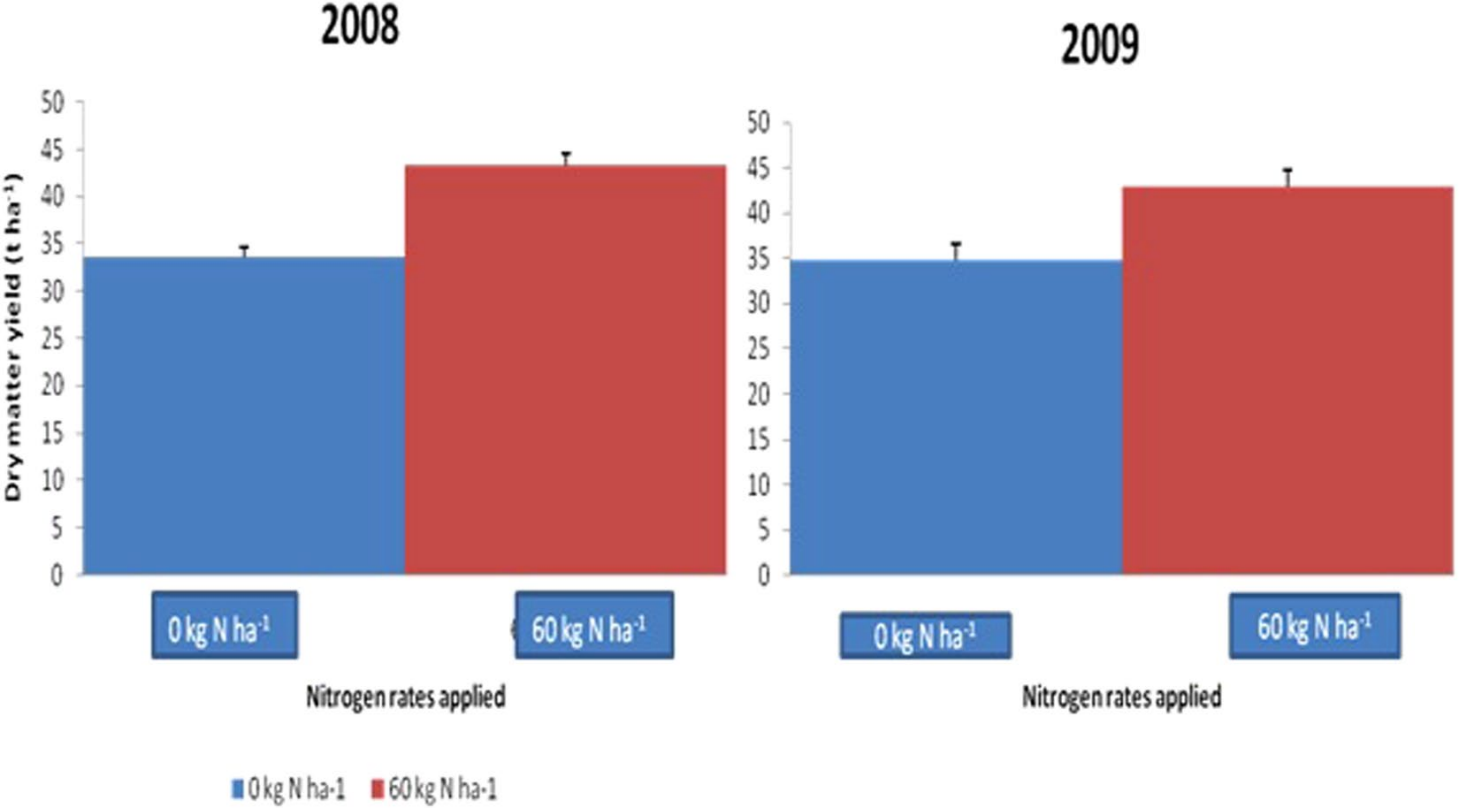
Effect of nitrogen application on maize dry matter yield in 2008 and 2009.

**Figure 5:**
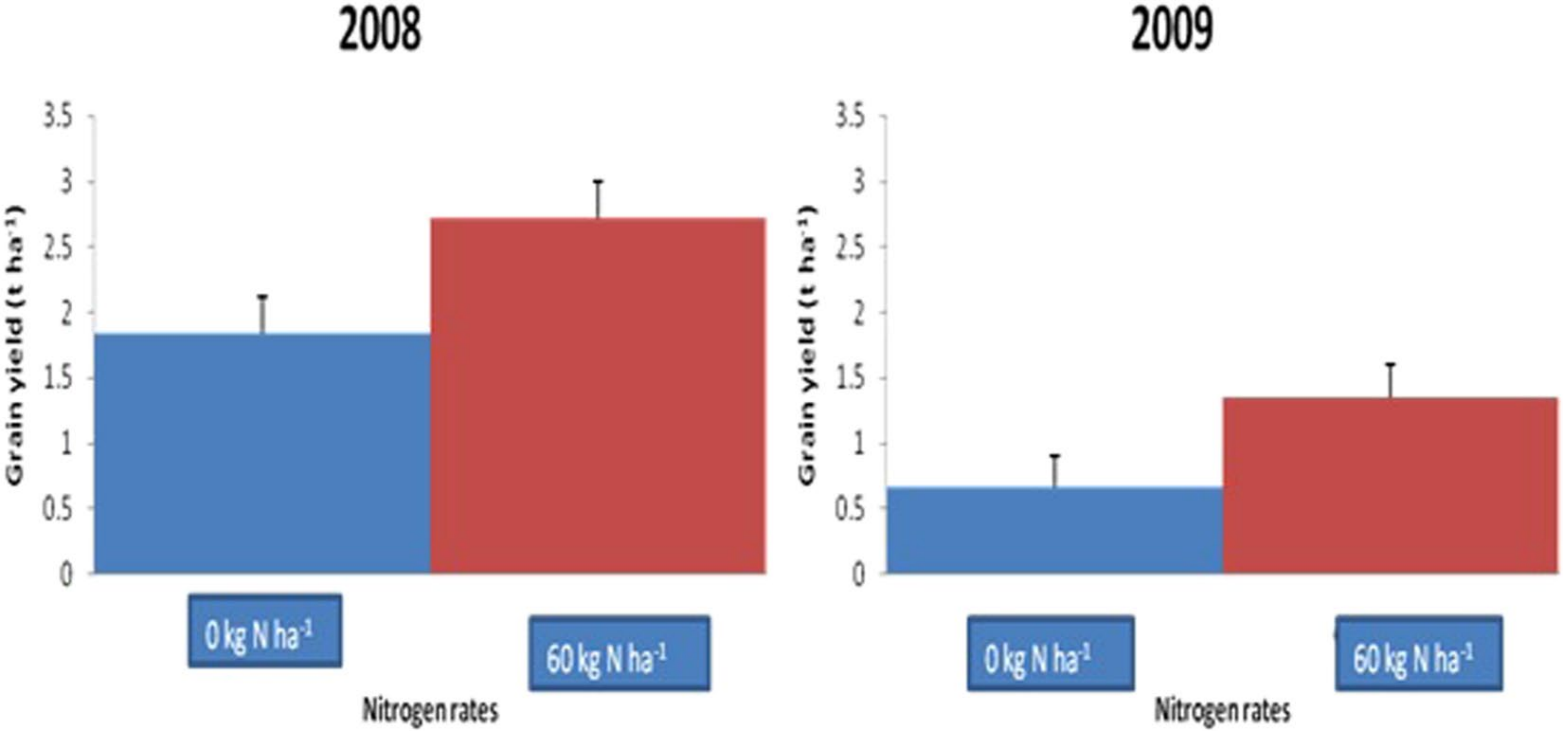
Effect of nitrogen application on maize grain yield in 2008.

Residue incorporation and fertilizer N application (60 kg ha^−1^) gave higher maize yield (2.74 tha^−1^ and 2.72 tha^−1^grain yield) over residue not incorporated (1.83 tha^−1^) and zero N application (1.84 tha^−1^) (Table 4, Figs 4 and 5). Table 5 shows that benefit resulting from the rotation was higher in plots with the residues of the first crop incorporated though cropping systems and residue management interaction significantly increased subsequent maize yield only in 2008. When compared with maize residue incorporation, velvet bean residue gave the highest rotation benefit (Table 6) contributing about 130% increase in maize yield over maize residue. This was followed by cowpea residue (10%) and least was soybean residue (2%).

**Table 5:** Effect of interaction of crop rotation systems and residue management on maize dry matter and grain yield.

Treatment combination	Dry matter (t ha^−1^)		Grain yield (tha^−1^)	
	2008	2009	2008	2009
Velvet bean-Maize × R I	52.28	49.95	4.66	1.90
Velvet bean-Maize × RNI	51.33	45.23	2.83	1.32
Cowpea-maize × RI	41.08	41.19	2.21	0.85
Cowpea-maize × RNI	38.27	38.07	1.87	0.59
Soybean-Maize × RI	40.99	38.01	2.07	0.95
Soybean-Maize × RNI	29.93	30.86	1.65	0.95
Maize-Maize × RI	33.74	38.71	2.02	0.93
Maize-Maize × RNI	20.54	28.54	0.97	0.55
LSD(0.05)	2.44**	ns	0.49**	ns

*significant at 5%, **highly significant at 1%, ns- not significant.

Note: RI means residue incorporated and RNI residue not incorporated.

**Table 6:** Percentage increase in maize yield of legume-maize plots over maize-maize plots (rotation benefit) with or without residue.

Treatments	2008	2009
Velvet bean-maize	147%	129%
Cowpea-maize	33%	NA
Soybean-maize	27%	43%
Velvet bean-Maize*Residue	130%	111%
Cowpea-maize*Residue	10%	NA
Soybean-Maize*Residue	2%	11%

Note: NA not applicable (no rotation benefit).

Residue production by preceding crop is an important factor in defining the magnitude of rotation benefit conferred to subsequent crop^13^. This is because Legume crop residues contain organic N and other nutrients, which are released after decomposition by soil microbes for the subsequent crop through mineralization^8^. And the quality of residue is very important in determining mineralization and nutrient release and then yield. Some of the residue parameters include C/N ratio, nutrient content etc. Maize residues contained more carbon and less nitrogen than velvet-bean residues and mineralization and release of nutrients were higher in plots amended with velvet-bean residue.

## Conclusion

Result of this research shows that there was increase in maize yield by legume-maize rotation over maize-maize rotation. Legume-maize rotation exerted significant increase on the maize component over maize-maize rotation. Comparing the different legume-maize rotations, increase in subsequent maize yield as a result of the rotation varied among the legume crops in rotation with maize. The degree of the rotation benefit was determined by the legume type and climatic condition at the time of growth, which affected the biomass production. Velvet bean-maize rotation increased maize yield producing significantly over 100% increase when compared with maize-maize rotation. Legume-cereal rotations significantly increased total soil nitrogen, exchangeable K, Mg and cation exchange capacity with variations in each of the years. There were also significant interaction effects between the legume-cereal rotations and their residue addition. Therefore, grain- and velvet bean-maize rotation cropping could be incorporated into the cropping system because it not only increased maize yield, it also improved the fertility potential of the soil.
